# Virtual Reality as an Adjuvant Treatment for Acute Pain During an Interventional Process with Capsaicin: A Feasibility Study

**DOI:** 10.3390/jcm14103590

**Published:** 2025-05-21

**Authors:** Anna Server, Maria Sonsoles Cepeda Diez, Carlos Suso-Ribera, Sara Guila Fidel Kinori, Diana Castilla, Francisco Javier Medel, Azucena García-Palacios

**Affiliations:** 1 Pain Unit, Vall d’Hebron University Hospital, 08035 Barcelona, Spain; anna.server@vallhebron.cat (A.S.); franciscojavier.medel@vallhebron.cat (F.J.M.); 2 Psychiatry Unit, Vall d’Hebron University Hospital, 08035 Barcelona, Spain; mariasonsoles.cepeda@vallhebron.cat (M.S.C.D.); sgfidel@gmail.com (S.G.F.K.); 3 Department of Psychiatry and Legal Medicine, Autonomous University of Barcelona, 08035 Barcelona, Spain; 4 Psychology Department, Universidad Europea, 38300 Canarias, Spain; 5 Rheumatology Unit, Vall d’Hebron University Hospital, 08035 Barcelona, Spain; 6 Department of Personality, Evaluation and Psychological Treatment, Universidad de Valencia, 46010 Valencia, Spain; diana.castilla@uv.es; 7 Department of Basic and Clinical Psychology and Psychobiology, Universitat Jaume I, 12006 Castellon de la Plana, Spain; azucena@uji.es; 8 CIBER Fisiopatología Obesidad y Nutrición (CIBERObn), Instituto Salud Carlos III, 28029 Madrid, Spain

**Keywords:** virtual reality, neuropathic pain, capsaicin, pain management, patient acceptability

## Abstract

**Background/Objectives**: This feasibility study explores the use of virtual reality (VR) as an adjunct therapy during capsaicin administration for individuals with chronic neuropathic pain. Chronic neuropathic pain poses significant management challenges due to its complex biopsychosocial nature. This study aimed to assess the acceptability, usability, and preliminary effectiveness of VR in reducing pain, anxiety, aversiveness, and rumination during painful procedures. **Methods**: A total of 24 patients participated in the study and received either capsaicin treatment with VR (n = 12) or treatment as usual (n = 12). The VR group engaged with “SnowWorld”, an immersive, interactive environment designed to promote distraction and relaxation. Outcomes including pain (average and worst), aversiveness, rumination, and anxiety, were assessed via 11-point scales. **Results**: Participants in the VR condition reported significantly lower scores for worst pain (2.83 vs. 6.33), average pain (2.08 vs. 5.42), aversiveness (1.50 vs. 6.08), rumination (1.17 vs. 5.75), and anxiety (0.83 vs. 5.17) compared to the controls (all *p* < 0.001). Participants reported high satisfaction regarding the VR experience, noting its immersive nature and ease of use. The qualitative feedback highlighted the VR’s ability to foster relaxation and distraction during capsaicin administration. **Conclusions**: These findings support the feasibility and preliminary efficacy of VR as an adjunctive tool for acute pain management during capsaicin treatment. Further studies with larger samples are warranted to confirm these effects and explore long-term outcomes.

## 1. Introduction

Pain is a complex perceptual process influenced by various biopsychosocial factors, including sensory, cognitive, behavioral, and emotional elements such as attention, memory, mood, emotion, patient expectations, motivation, and context [1,2]. Neuropathic pain (NP), which results from nerve injury, presents a major challenge for clinicians in Pain Units due to its pervasive nature and high global prevalence [3,4]. Efforts to enhance the range of treatments for neuropathic pain have led to the increased popularity of interventional techniques, which are becoming more prominent compared to traditional pharmacological approaches, as reflected in recent treatment algorithms [5].

In particular, capsaicin patches show great potential and are increasingly being used for the management of chronic neuropathic pain [6]. However, their administration requires a hospital setting due to the frequent occurrence of side effects, such as airway and mucous membrane irritation (e.g., erythema), allergic reactions, itching, stinging, burning, acute pain, and increased blood pressure [7,8]. Aligned with the biopsychosocial perspective of pain, various non-pharmacological interventions have been used as adjuncts in the management of acute pain [9,10]. For over three decades, virtual reality (VR) has emerged as an increasingly popular approach for managing acute painful procedures, gradually replacing other distraction techniques that rely on external focus due to its appeal, immersive experience, and increasingly affordable cost [11,12].

VR is a technology that creates a computer-generated virtual world, providing users with an immersive and interactive experience. Using special glasses and a device that tracks head movements, users feel as if they are inside a virtual environment that includes various sensory elements and responds to their movements similarly to the real world [13]. A growing body of evidence supports the efficacy of VR in managing acute pain across various populations and procedures, including both children and adults [14,15,16]. Recent studies also suggest that VR may play a significant role in reducing pain intensity in chronic pain patients, further solidifying its potential as a valuable tool in pain management [17].

The mechanism by which VR enhances the subjective experience of pain is not yet fully understood. It is believed that distraction and the resulting pain reduction stem from the patient’s engagement in a virtual environment filled with artificially generated stimuli [18,19]. VR creates an artificial, dynamic, three-dimensional environment that enhances attention load by incorporating visual, auditory, and proprioceptive inputs, making it more cognitively engaging than traditional distraction methods like music or guided imagery [20,21]. In the absence of competing stimuli, attention tends to be monopolized by pain [22]. Therefore, a potential mechanism for reducing incoming pain and anxiety signals before and during procedures is the redirection of attentional resources [19], a process that is enhanced with the sense of presence or immersion [20].

Beyond attentional mechanisms, neuroimaging studies have begun to reveal how VR modulates pain-related brain activity. For instance, VR has been shown to reduce activity in regions such as the anterior cingulate cortex, insula, and thalamus—areas consistently implicated in pain perception and affective processing—while enhancing activation in the prefrontal cortex, which plays a role in top-down modulation of pain [23]. This suggests that VR not only distracts but may also engage cortical networks responsible for cognitive control and emotion regulation, thereby altering pain appraisal at multiple levels of processing. Therefore, compared to simpler interventions like guided imagery or passive music listening, VR’s interactive and immersive features might offer a more robust engagement of these neural pathways, potentially leading to greater analgesic effects. Consequently, VR has been proposed as a useful adjunctive therapy for painful procedures [24].

Managing acute pain during traditional methods remains challenging. Given the growing evidence supporting the effectiveness of VR in these contexts, along with reduced costs, enhanced quality of technology (e.g., more portable and lightweight options), and better tolerance regarding cybersickness [25,26], the introduction of VR in acute care management and rehabilitation has been recommended [27]. There is preliminary but promising evidence suggesting that VR can help reduce some potential adverse effects of capsaicin treatments in healthy individuals, such as ongoing pain and secondary hyperalgesia [28]. However, the extent to which these findings from a community sample apply to individuals with chronic neuropathic pain, where peripheral and central sensitization influence pain transmission and interpretation [29], is unclear.

This feasibility study builds on previous research by examining the utility of VR during capsaicin administration for individuals with chronic neuropathic pain, focusing on outcomes beyond pain relief, consistent with a biopsychosocial approach to pain. Given the absence of prior studies applying VR in this context, we designed this as a pilot trial with a small sample to evaluate feasibility—defined by reach and acceptability—and to explore preliminary effects on the aforementioned outcomes, which might also serve for future sample size calculations in larger trials. Our main aim, however, was not to establish efficacy, but rather to assess practical implementation in a clinical setting and guide future randomized controlled trials. Drawing from the findings of Caserman et al. [25], who reviewed the occurrence of cybersickness in current VR headsets, we also prioritized the evaluation of potential side effects to better understand the therapeutic feasibility of VR in real-world environments.

Caserman et al.’s findings on cybersickness indicate that although VR-related side effects remain a concern, advancements in technology are minimizing these occurrences, suggesting that any side effects will likely be infrequent and manageable [25]. Accordingly, we hypothesize that most participants will tolerate VR with minimal adverse effects and will find it user-friendly and acceptable. Furthermore, we anticipate that a majority of potential participants assigned to the VR condition will be open to using the tool (reach). Lastly, we expect that the VR group will report reductions in pain, anxiety, disgust, and rumination compared to those receiving standard treatment without VR, highlighting VR’s therapeutic potential in managing multifaceted aspects of chronic pain.

## 2. Materials and Methods

### 2.1. Procedures

This is a feasibility study with two conditions. Both groups received capsaicin treatment for their pain, but the experimental group used VR during the administration of the treatment (VR condition), while the comparison group followed the standard procedure without VR (treatment as usual, or TAU condition). Potential participants included consecutive patients with peripheral neuropathic pain (PNP), identified according to the established criteria from the International Association for the Study of Pain [30], attending the Pain Clinic at Vall d’Hebron Hospital in Spain. The clinical team screened all patients at the Pain Clinic from January 2022 to September 2022 for preliminary eligibility based on the type of pain justifying capsaicin treatment and their physical/mental status. Patients who agreed to participate were then assigned to a condition following the randomization order from a list created by an external researcher using an online randomization tool.

Inclusion criteria included the presence of peripheral neuropathic pain that warranted capsaicin treatment, the physical and mental/cognitive ability to use the VR application, and a willingness to participate, accompanied by signing the informed consent. While there was no formal upper age limit, all participants were adults aged 18 or older, as is standard in the Pain Clinic’s patient population. Exclusion criteria included any physical impairments that might interfere with the ability to use the VR equipment (e.g., wounds or bandages on the head, infections, visual or auditory problems), as well as severe mental or cognitive alterations such as dementia, intellectual disabilities, severe and active psychiatric illnesses (e.g., schizophrenia with active hallucinations or delusions, or active substance abuse), and clinical confusion. Patients with a medical history of cerebrovascular accidents, seizures, epilepsy, motion sickness, vertigo, nausea, or active vomiting were also excluded to prevent complications with VR. For ethical reasons, individuals who were part of the study team, family members of the study team, or subordinates or direct family members of a subordinate of the staff involved in the study were excluded.

Given the feasibility focus of the study, we focused on the immediate or acute effects of VR during capsaicin administration, rather than long-term outcomes. Assessing short-term responses allowed us to evaluate the acceptability, tolerability, and initial effectiveness of the VR intervention in a real-world clinical context. This focus is consistent with the study’s goal of determining whether VR can be practically implemented as an adjunctive tool during a known painful procedure, thereby informing the design of future efficacy trials.

All study procedures were approved by the ethics committee of Vall d’Hebron Hospital (Barcelona, Spain) under protocol number PR(ATR)338/2021.

### 2.2. Measures

#### 2.2.1. Primary Outcomes

This study includes feasibility measures of reach and acceptability of the VR tool, along with preliminary data on the effectiveness of the VR system in reducing pain, disgust, anxiety, and rumination. Reach, defined as the proportion of individuals who can effectively use the tool from all potential candidates, will be evaluated as a percentage. Acceptability will be assessed using four items that address ease of use (“How EASY was it to use the VR?”), side effects (“To what extent did you feel NAUSEA when using the VR?”), immersion (“To what extent did you feel like you were INSIDE the virtual world?”), and enjoyment (“How FUN was it to use Virtual Reality (VR)?”). Response options for these items will range from 0 = “Not at all” to 10 = “Very much”. Additionally, two open-ended questions will be included to evaluate what participants liked the “Most” and “Least” about the VR experience.

Preliminary effectiveness outcomes will assess the experience of pain, including average and maximum severity, as well as disgust and rumination during capsaicin administration. For all five constructs, we will use an 11-point numerical verbal scale ranging from 0 = “no pain” to 10 = “maximum pain”. The items are based either on the Spanish validation of the Brief Pain Inventory [31] or on previous research conducted by our group utilizing single items [32].

#### 2.2.2. Secondary Outcomes

Secondary outcomes will include measures of baseline pain status (Brief Pain Inventory, BPI) [31], health-related quality of life (EuroQoL-5d-5L) [33], depression and anxiety (Hospital Anxiety and Depression Scale, HADS) [34], and catastrophizing (Pain Catastrophizing Scale, PCS) [35] prior to capsaicin administration.

The BPI evaluates pain severity, including average, minimum, and maximum pain over the last 24 h, as well as current pain, and assesses how pain interferes with seven areas of daily functioning. Items use an 11-point Likert scale ranging from 0 = “No pain/interference” to 10 = “Pain as bad as you can imagine/Maximum interference”. The EuroQoL-5D-5L is a brief, 5-item scale measuring health-related quality of life, focusing on difficulties related to mobility, daily activities, self-care, pain/discomfort, and anxiety/depression. A total score is calculated by summing all items, with higher scores indicating poorer health-related quality of life.

The HADS consists of 14 items, with 7 assessing anxiety and 7 assessing depressive symptoms. A 4-point Likert scale ranging from 0 = “not at all” to 3 = “most of the time” is used, with higher scores indicating more severe depression or anxiety symptoms. Finally, the PCS evaluates catastrophic thinking about pain with 13 items, where responses range from 0 = “not at all” to 4 = “all the time”, with higher scores reflecting greater catastrophizing.

### 2.3. Interventions

#### 2.3.1. Medical Intervention

The capsaicin 8% patch (Qutenza^®^ (Grünenthal GmbH, Aachen, Germany)) was commercialized for hospital administration 10 years ago. Capsaicin, a substance derived from a type of chili, initially produces a local irritation effect followed by prolonged loss of sensitivity [7]. Due to the properties of the capsaicin medication, it must be administered by a healthcare professional; self-administration by the patient is not permitted. The treatment requires a controlled environment and protective measures for the administrator, such as gloves and glasses. It is administered in a single-use box, with patients receiving the drug alone during the one-hour administration period. Both treatment conditions in this study received the same capsaicin treatment for their pain.

Capsaicin has demonstrated high effectiveness, especially in the short term [36,37]. However, due to the characteristics of the substance, the use of this patch may cause local burning, pain, erythema, and itching during application. Although these adverse effects typically subside upon removal of the patch, some patients may require a topical anesthetic to alleviate the acute pain caused by the substance, as well as cold compresses and oral analgesics afterward.

#### 2.3.2. Virtual Reality

Although the need to remove the capsaicin patch and interrupt treatment due to side effects is low, at 0.8% [38,39], the experience can be very unpleasant for some patients, negatively impacting the prevalence of its administration [8]. To mitigate this unpleasantness, an adjunctive VR intervention was implemented during capsaicin administration in the VR condition of this study, given its proven ability to induce distraction in similar contexts, such as burn care [12] and skin itch problems [21].

The VR session lasted approximately 60 min, corresponding to the full duration of capsaicin patch administration. The VR environment used was SnowWorld, a widely validated virtual setting designed specifically for immersive distraction in painful contexts [40,41,42]. SnowWorld is a 3D interactive environment featuring a cold, snowy landscape, populated with igloos, penguins, snowmen, and fish, counteracting the sensations of heat, stinging, burning, and pain that may arise from medical procedures, including the application of the capsaicin patch. Unsurprisingly, SnowWorld has become the most widely used virtual environment for immobilized burn patients [12].

Patients could navigate visually through the environment and interact with it by throwing snowballs at various animated elements, creating a playful and engaging distraction. These interactions were facilitated via a mouse controller, and the experience was enhanced with ambient sound effects and background music consistent with the snowy setting, aiming to evoke a sense of coolness and calm that contrasts the burning sensation caused by capsaicin [43]. Research supports the relevance of using immersive and interactive VR environments like SnowWorld in improving pain management outcomes [44].

**Figure 1:**
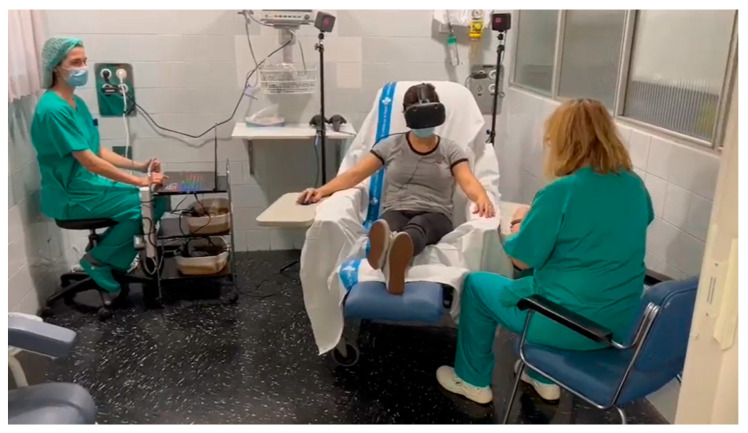
Example picture of the VR-enhanced capsaicin treatment.

For VR visualization, patients used HTC Vive glasses (HTC Corporation, Taiwan, China) and a mouse to interact with the virtual environment. The VR device complies with the R&TTE Directive (1999/5/EC) issued by the European Commission. See Figure 1 for an illustration of the procedure.

To reduce any risk of adverse effects like motion sickness or disorientation, the scenario involved minimal camera motion, and users remained stationary, reducing vestibular conflicts that might contribute to cybersickness. Patients could take breaks if needed, although none requested to interrupt the session apart from one individual who discontinued due to headache.

## 3. Results

Following randomization and group assignment, we evaluated the feasibility of the VR intervention. The reach of the study was 100%, as all eligible patients who were approached agreed to participate. Within the VR condition, 92.3% (12 out of 13) of randomized participants were able to effectively use the VR system, indicating high usability. One participant experienced headaches and declined to use the system after initial exposure.

In terms of participant characteristics, the final sample comprised 24 participants, with 12 randomly assigned to the VR condition and 12 to the treatment-as-usual condition. The sociodemographic characteristics of the two groups were comparable. In the VR condition, the mean age was 57.0 years (SD = 10.5), 75.0% were women, and none of the participants were actively working at the time of assessment, as most were either on sick leave or homemakers. In the treatment-as-usual group, the mean age was 55.9 years (SD = 13.7), 66.7% were women, and only one participant was actively working at the time of assessment; again, the majority were either on sick leave or homemakers.

Regarding the clinical characteristics of the two groups, the results for the VR condition were as follows: pain in the last 24 h (mean = 5.8, SD = 2.2), catastrophizing (mean = 33.6, SD = 8.2), quality of life (mean = 9.5, SD = 1.8), anxiety (mean = 8.9, SD = 3.1), and depression (mean = 7.3, SD = 3.8). In the treatment-as-usual condition, the baseline characteristics were as follows: pain in the last 24 h (mean = 6.3, SD = 1.5), catastrophizing (mean = 37.6, SD = 6.7), quality of life (mean = 9.4, SD = 1.2), anxiety (mean = 8.7, SD = 3.5), and depression (mean = 6.5, SD = 4.0). Analyses of group differences at baseline for all clinical characteristics indicated that both conditions had comparable characteristics before the experimental study (all Mann–Whitney tests were *p* > 0.05). Corresponding effect sizes were small to moderate, with Cohen’s d values of −0.27 for pain, −0.53 for catastrophizing, 0.07 for quality of life, 0.06 for anxiety, and 0.21 for depression.

VR exposure lasted the full 60 min during capsaicin administration, with no interruptions. Regarding the usability of the tool (on a 0–10 scale), the average enjoyment (fun) reported during the VR game while receiving capsaicin treatment was 8.5 (SD = 1.5). The average ease of use of the VR tool was 8.6 (SD = 1.7), and the average immersion level was 7.5 (SD = 2.0). Cybersickness, including symptoms like nausea or dizziness, was rare. The mean nausea score was 0.3 (SD = 1.2), and no participants requested early removal of the headset due to cybersickness. This suggests a high tolerability of the VR intervention in this patient population.

When participants were asked what they liked most about the tool, their responses revealed three overarching themes: relaxation and emotional relief (e.g., “relaxation”, “frozen panoramic view”; n = 4), distraction and engagement (e.g., “distraction”, “everything”; n = 5), and design and entertainment value (e.g., “the little character design”, “fun”; n = 3). These themes, summarized in Supplementary Table S1, underscore the perceived emotional and attentional benefits of the VR experience, as well as its engaging aesthetic qualities.

Regarding aspects they liked least, comments included the weight of the VR glasses (n = 6), image quality at certain points (n = 2), dizziness (n = 1), and the childish nature of the content (n = 1). Two patients stated that they had no negative feedback.

Regarding the preliminary effectiveness of the VR tool, Mann–Whitney tests indicated significant results favoring the VR condition for all outcomes experienced during the procedure: worst pain (Z = −4.02, *p* < 0.001, r = −0.82), average pain (Z = −4.21, *p* < 0.001, r = −0.86), aversiveness (Z = −4.19, *p* < 0.001, r = −0.85), pain rumination (Z = −4.17, *p* < 0.001, r = −0.85), and anxiety (Z = −3.85, *p* < 0.001, r = −0.79). These represent large effect sizes (all r > 0.75). The means and standard deviations of these outcomes for both conditions are presented in Table 1.

## 4. Discussion

This feasibility study aimed to explore the utility of virtual reality during capsaicin administration in individuals with chronic neuropathic pain. The results indicate promising outcomes regarding the acceptability, usability, and preliminary effectiveness of VR as a distraction technique in managing acute pain associated with medical procedures. By examining participants’ experiences in a real clinical environment, this study not only contributes to the existing literature on pain management but also offers valuable insights into how VR can serve as a beneficial adjunct therapy for individuals suffering from chronic pain.

Our findings revealed that the use of VR significantly reduced various pain-related outcomes compared to the treatment-as-usual condition. Participants in the VR group reported lower levels of worst pain, average pain, aversiveness, pain rumination, and anxiety during capsaicin application. These results are consistent with previous evidence supporting VR’s effectiveness in procedural pain management, such as in burn care [45], and extend its application to chronic neuropathic pain—a less commonly explored area.

The observed analgesic effects likely reflect VR’s capacity to redirect attentional and emotional resources away from nociceptive input, consistent with the gate control theory of pain [46]. Compared to traditional distraction methods such as music or guided imagery, VR’s immersive environments might engage multiple sensory channels and require greater cognitive involvement [21,47], potentially contributing to its stronger analgesic potential. The use of the SnowWorld environment, which visually contrasts with the heat-induced pain of capsaicin through cold-toned, ice-themed imagery, may enhance this effect. Prior studies have shown that sensory incongruence between visual input and thermal stimuli can modulate pain perception arguably via top-down neural processes [40]. This targeted use of visual context supports the theoretical rationale behind scene selection. Nonetheless, future studies should compare different VR content types (e.g., natural landscapes, cognitive-behavioral VR) to evaluate their relative effects on pain, emotional state, and treatment adherence.

Participants rated the VR experience as highly enjoyable and immersive—factors known to enhance pain relief outcomes [43]. These user-centered aspects are critical to the feasibility and acceptability of VR in clinical practice [44,48]. While distraction remains a key mechanism, our findings add to emerging research suggesting that VR’s benefits may also involve modulation of neural pain pathways and emotional regulation—mechanisms warranting further exploration in head-to-head comparisons with other non-pharmacological interventions.

Regarding safety, side effects were minimal: only one participant dropped out and another reported mild nausea (rated 0.3/10). Although this suggests good tolerability, it is important to note that the intervention was limited to a single, brief session of approximately one hour. This short duration might have minimized the risk of adverse effects and limits the generalizability of our safety findings to longer-term or repeated use. While engagement was not a problem during the VR sessions, concerns about potential overuse and dependency with prolonged exposure remain relevant [49]. Future studies should include follow-up assessments to detect signs of withdrawal or compulsive use.

Beyond pain outcomes, we observed significant reductions in anxiety and pain rumination, both of which are clinically relevant given their strong links to chronic pain [50]. Chronic pain patients often experience elevated anxiety, which can intensify pain perception and reduce treatment engagement [51]. By promoting engagement and psychological relief, VR may disrupt this negative cycle and improve the overall treatment experience. Importantly, these psychological benefits occurred in a high-stimulus context, reinforcing VR’s potential to support emotional regulation during intense pain episodes.

Moreover, the inclusion of qualitative feedback from participants further enriched our understanding of the user experience. Many participants reported feeling relaxed and distracted during the VR session, indicating that the technology not only served to mitigate pain but also fostered a sense of well-being during the capsaicin administration. Thematic analysis revealed three dominant areas of appreciation: relaxation and emotional relief, distraction and immersive engagement, and aesthetic enjoyment of the design. Overall, these findings align with findings from prior studies indicating that VR can enhance emotional regulation and attentional redirection during painful experiences, ultimately leading to improved patient satisfaction and compliance [52]. Participants’ feedback on what they liked and disliked regarding the VR experience also highlighted areas for potential improvement, such as optimizing the comfort of VR equipment and enhancing image quality. Addressing these aspects could further increase the feasibility and appeal of VR interventions in clinical settings.

Despite the promising findings, our study has several limitations. First, the small sample size (n = 24), though appropriate for a feasibility study, limits statistical power, precludes subgroup analyses, and reduces generalizability. Consequently, the results should be interpreted as preliminary. No a priori sample size calculation was conducted due to the exploratory nature of the trial; however, based on observed effect sizes (r = 0.79 to 0.83), we estimate that 34–46 participants would be required to detect large effects (d ≈ 1.0), and up to 128 for medium effects (d = 0.5) in a fully powered trial. Recruitment was limited by the specific clinical context and single-center design. Future multicenter studies could help overcome these barriers and increase statistical precision.

Second, although baseline comparisons were not statistically significant, small-to-moderate effect sizes for catastrophizing (d = −0.53) and pain (d = −0.27) indicate possible confounding. Future studies should consider covariate adjustment (e.g., ANCOVA) for significant or large effect sizes to account for baseline differences and assess potential moderators such as age, pain duration, and psychological characteristics. Another limitation is the focus on a single, short-term intervention. While this allowed for the assessment of immediate outcomes, it precluded the evaluation of longer-term benefits. Future research should include follow-up at 1–3 months to examine whether the effects of VR on pain, anxiety, and rumination are sustained and whether it enhances willingness to repeat painful treatments like capsaicin. Monitoring for signs of habituation, withdrawal, or overuse may also clarify the risk of VR dependence in therapeutic contexts.

Finally, economic feasibility should also be considered. Although the VR system used in this study may not be readily available in all settings, lower-cost, commercially available headsets are becoming increasingly accessible [53]. Future research should evaluate the cost-effectiveness of VR interventions and explore scalable models for clinical implementation.

## 5. Conclusions

In conclusion, this study adds to the growing body of evidence supporting the use of VR as an effective adjunct therapy for managing pain and anxiety in patients with chronic neuropathic pain. The results demonstrate that VR can enhance the treatment experience, promoting both pain relief and psychological well-being. Given the positive feedback regarding the acceptability and usability of the VR tool, there is a compelling case for its routine implementation in clinical practice. As healthcare continues to evolve, integrating innovative technologies such as VR into pain management protocols may pave the way for more effective and patient-centered approaches to treating chronic pain conditions.

Future studies should build upon these findings by exploring the long-term efficacy of VR interventions, expanding the patient population, and addressing the limitations identified in this study. By continuing to investigate the potential of VR in pain management, we can work toward enhancing the quality of life for individuals living with chronic pain.

## Figures and Tables

**Table 1:** Descriptive values and analysis of group differences of outcomes during capsaicin administration.

		Mean (SD)	Z-Value	*p*-Value
Worst pain	VR Condition	2.83 (1.12)	−4.02	<0.001
	Treatment as Usual	6.33 (1.30)		
Average pain	VR Condition	2.08 (0.79)	−4.21	<0.001
	Treatment as Usual	5.42 (1.44)		
Aversiveness	VR Condition	1.50 (1.09)	−4.19	<0.001
	Treatment as Usual	6.08 (1.51)		
Rumination	VR Condition	1.17 (1.19)	−4.17	<0.001
	Treatment as Usual	5.75 (1.77)		
Anxiety	VR Condition	0.83 (1.60)	−3.85	<0.001
	Treatment as Usual	5.17 (2.04)		

## Data Availability

Data will be accessible upon request to the corresponding author.

## Supplementary Materials

The following supporting information can be downloaded at: https://www.mdpi.com/article/10.3390/jcm14103590/s1.

## Abbreviations

The following abbreviations are used in this manuscript:

VR	Virtual Reality
TAU	Treatment As Usual
BPI	Brief Pain Inventory
HADS	Hospital Anxiety and Depression Scales
PCS	Pain Catastrophizing Scale
