# Supplementary material for: Virtual Reality as an Adjuvant Treatment for Acute Pain During an Interventional Process with Capsaicin: A Feasibility Study
Source: J Clin Med. 2025 May 21;14(10):3590. doi: 10.3390/jcm14103590 (PMC12112111; doi:10.3390/jcm14103590)
Supplement: Supplementary file 1 [file jcm-14-03590-s001.zip › jcm-3630592-supplementary.pdf]

Supplementary Table S1: Feedback Themes about Virtual Reality

Theme	Description	Example Responses	Number of Mentions (n = 12)
**Relaxation and Emotional Relief**	Feelings of calm, serenity, and reduced anxiety during VR use	"Relaxation", "Frozen panoramic view"	4
**Distraction and Engagement**	Perceived redirection of attention away from pain or procedure	"Distraction", "Everything"	5
**Design and Entertainment Value**	Appreciation of visual elements and entertainment features	"The little character design", "Fun"	3
